# Endoscopic Submucosal Dissection for Early Gastric Cancer in Elderly vs. Non-Elderly Patients: A Systematic Review and Meta-Analysis

**DOI:** 10.3389/fonc.2021.718684

**Published:** 2022-01-13

**Authors:** Jiting Zhao, Zhen Sun, Junwei Liang, Song Guo, Di Huang

**Affiliations:** ^1^ Department of Spleen and Stomach Disease, Shandong University of Traditional Chinese Medicine Affiliated Hospital, Jinan, China; ^2^ Shandong University of Traditional Chinese Medicine, Jinan, China

**Keywords:** gastric cancer, surgery, geriatric, complications, endoscopy

## Abstract

**Objective:**

This study aimed to review the applicability and complications rate associated with endoscopic submucosal dissection (ESD) for early gastric cancer in elderly patients.

**Methods:**

Databases of PubMed, Embase, CENTRAL, and ScienceDirect were searched till 15^th^ April 2021. All types of studies comparing ESD in the elderly vs non-elderly were included. Subgroup analysis was conducted for the following groups: ≥80 years vs <80 years, ≥75 years vs < 75 years, and ≥65 years vs <65 years.

**Results:**

17 studies were included. Meta-analysis indicated no statistically significant difference in the en-bloc resection rates (OR: 0.92 95% CI: 0.68, 1.26 I^2^ = 8% p=0.62) and histological complete resection rates (OR: 0.93 95% CI: 0.75, 1.15 I^2^ = 26% p=0.50) between elderly and non-elderly patients. The results were non-significant even on subgroup analysis. Overall, we found a non-significant but a tendency of increased perforation rates in the elderly as compared to non-elderly patients (OR: 1.22 95% CI: 0.99, 1.52 I^2^ = 0% p=0.06). However, there was a significantly increased risk of perforation in elderly patients aged ≥80 years as compared to patients <80 years (OR: 1.50 95% CI: 1.00, 2.24 I^2^ = 3% p=0.05). Bleeding rates were not different in the two groups (OR: 1.07 95% CI: 0.87, 1.32 I^2^ = 19% p=0.52). Pooled analysis indicated a statistically significantly increased risk of pneumonia in elderly patients (OR: 2.52 95% CI: 1.72, 3.70 I^2^ = 7% p<0.00001). Length of hospital stay was reported only by five studies. Meta-analysis indicated no significant difference between the two study groups (MD: 0.67 95% CI: -0.14, 1.48 I^2^ = 83% p=0.10).

**Conclusion:**

En-bloc and histological complete resection rates do not differ between elderly and non-elderly patients undergoing ESD for early gastric cancer. Elderly patients have a small tendency of increased risk of perforation with significantly increased rates in the super-elderly (≥80 years of age). The risk of pneumonia is significantly higher in elderly patients but the rates of bleeding do not differ. The certainty of evidence is “very low” and there is a need for high-quality studies taking into account confounding factors to enhance the quality of evidence.

## Introduction

Over the years, there has been a gradual improvement in life expectancy owing to significant advances in healthcare and accessibility of medical resources worldwide. Almost every country in the world is experiencing an increase in the proportion of elderly individuals in their overall population. According to the 2019 United Nations report, around 703 million persons were above the age of 65 years in 2019 and this figure is expected to double to 1.5 billion by 2050 (1). As healthcare professionals are expected to face an increased load of elderly patients in their practice, the efficacy and safety of different surgical procedures must be optimally understood for these individuals.

Gastric cancer is one of the leading causes of death, especially in older adults. It is estimated to the 5^th^ most common cancer and third most lethal malignancy causing around 783,000 deaths in 2018 alone (2). Epidemiological data indicate that gastric cancer is highly prevalent in regions of East Asia, eastern Europe, and Russia (3). With a rise in the incidence of esophagogastroduodenoscopy in elderly patients, a large number of patients with early gastric cancer are frequently detected in these regions (4). Over the last few decades, endoscopic submucosal dissection (ESD), a minimally invasive technique, has become the standard treatment modality for the management of early gastric cancer (5). As gastric cancer in the early stages is confined to the superficial layers of the mucosa, endoscopic dissection can be safely performed to excise the entire lesion for histopathological evaluation, thereby minimizing patient morbidity and mortality (6). The procedure consists of an initial injection of fluid in the submucosal layer to elevate the lesion. This is followed by a circular incision on the surrounding mucosa of the lesion and subsequent dissection of the submucosal layer to completely elevate the tumor (7). Studies have demonstrated that ESD is safe and feasible in patients with early gastric cancer with comparable long-term survival as compared to gastrectomy (8).

Since elderly patients have poor overall health status along with several other comorbidities, ESD is an attractive treatment option in these patients as compared to gastrectomy to minimize operative morbidity. However, it is not very clear if ESD per-se safe and feasible in this group of patients. It is important to know if the resection rates in elderly patients are comparable to non-elderly patients to recommend it as a treatment option. Furthermore, clinicians should have a clear understanding of the risk of complications with ESD in this cohort so that appropriate preventive measures can be taken to reduce them. To the best of our knowledge, to date, only one meta-analysis published in 2015 has assessed outcomes of ESD in the elderly, but it could include only nine studies (9). The review was also unable to differentiate outcomes based on various definitions of elderly (≥65 years, ≥75 years, or ≥80 years). Further, with the publication of several recent studies (10–12), there is a need for updated evidence on the applicability and safety of ESD in elderly patients. In this context, the current study was designed to compare resection rates, complication rates, and length of hospital stay between elderly and non-elderly patients undergoing ESD for early gastric cancer.

## Material and Methods

### Search Strategy

The PRISMA statement (Preferred Reporting Items for Systematic Reviews and Meta-analyses) was followed during the conduct of this review (13). We searched for eligible studies electronically on the databases of PubMed, Embase, CENTRAL, and ScienceDirect. Two authors carried out the literature search independent of each other. The lower time limit of the search was from the inception of the databases. The last search was conducted on 15^th^ April 2021. Keywords used in various combinations were: “elderly”, “aged”, “older”, “geriatric”, “endoscopic submucosal dissection”, and “gastric cancer”. Details are provided in **Supplementary Table 1**. The results of each database were reviewed by their titles and abstracts and articles relevant to the review were segregated. The two authors evaluated the full text of these articles for final inclusion in the study. Any disagreements in the selection process were resolved by discussion. Finally, we also performed a hand-search of the bibliography of studies meeting the inclusion criteria and previous reviews on the topic for any missed references.

### Eligibility Criteria

The inclusion criteria of the review were outlined based on the PICO (Population, Intervention, Comparison, Outcome) guideline. We included studies which were:

1. All studies conducted on a *Population* of adult early gastric cancer patients undergoing ESD.
2. had a group (*Intervention*) of elderly patients (age group defined as per the study).
3. being *Compared* with a group of non-elderly patients.
4. reporting one of the following *Outcomes*:- en-bloc dissection rate, histological complete resection rates, length of hospital stay, or complications.

Exclusion criteria for the review were are follows: 1) Studies not on patients with early gastric cancer 2) Studies not defining “elderly” population 3) Single arm studies not comparing outcomes with non-elderly group 4) Non-English language studies, case reports, and review articles. 5) Studies reporting duplicate data. In case of two or more studies were from the same healthcare setup, we included the article with the largest sample size.

### Data Extraction and Quality Assessment

A data extraction form was prepared beforehand by the authors to extract relevant data. Information was sourced by two authors independently. Name of the first author, publication year, study type, study location, the definition of elderly, study groups, sample size, demographic details, comorbidity status (cardiovascular disease and diabetes), use of antithrombotic or anticoagulant drugs, ulcer finding, lesion location, lesion depth, histological type, tumor size, lymphatic invasion, and study outcomes were extracted.

The primary outcomes were en-bloc resection rates and histological complete resection rates. En-bloc resection was defined as resection of the tumor in one piece. Histological complete resection was defined as the histological identification of tumor-free margins in the resected tissue. Secondary outcomes were complications namely; incidence of perforation, bleeding, and pneumonia. Complications included both intraoperative and postoperative incidence combined. For the primary outcomes, data were pooled based on the number of lesions while for the secondary outcome, data were pooled based on the number of patients.

The methodological quality of included studies was assessed using the Newcastle-Ottawa scale (NOS) (14). This too was carried out in duplicate and independently by two study investigators. Studies were awarded points for selection of study population, comparability, and outcomes. The maximum score which can be awarded is nine. The certainty of the evidence was assessed using the Grading of Recommendations Assessment, Development, and Evaluation (GRADE) tool using the GRADEpro GDT software [GRADEpro Guideline Development Tool. McMaster University, 2020 (developed by Evidence Prime, Inc.)].

### Statistical Analysis

Meta-analysis was carried out using “Review Manager” (RevMan, version 5.4; Nordic Cochrane Centre [Cochrane Collaboration], Copenhagen, Denmark; 2014). On account of the inherent heterogeneity amongst the included studies, a random-effects model was used for the meta-analysis of all outcomes. Odds ratios (OR) with 95% confidence intervals (CI) were calculated to compare resection rates and complications between the elderly and non-elderly groups. Mean and standard deviation (SD) data of the length of hospital stay was extracted and pooled to calculate the mean difference (MD) and 95% CI. Heterogeneity was assessed using the I^2^ statistic. I^2^ values of 25-50% represented low, values of 50-75% medium, and more than 75% represented substantial heterogeneity. We used funnel plots to assess publication bias for the primary outcomes. Since the definition of elderly differed across included studies, we carried out a subgroup analysis for the variable definitions. We divided the data into the following three subgroups: ≥80 years vs <80 years, ≥75 years vs < 75 years, and ≥65 years vs <65 years. A sensitivity analysis was also performed for a meta-analysis of resection rates and complications. Individual studies were sequentially excluded from the meta-analysis in the software itself to check any undue influence of the study on the total effect size. P ≤ 0.05 was considered statistically significant. For analyses with I^2^<50%, we also checked the results using a fixed-effects model in the meta-analysis software for any change in the significance of the results.

## Results

**Figure 1:**
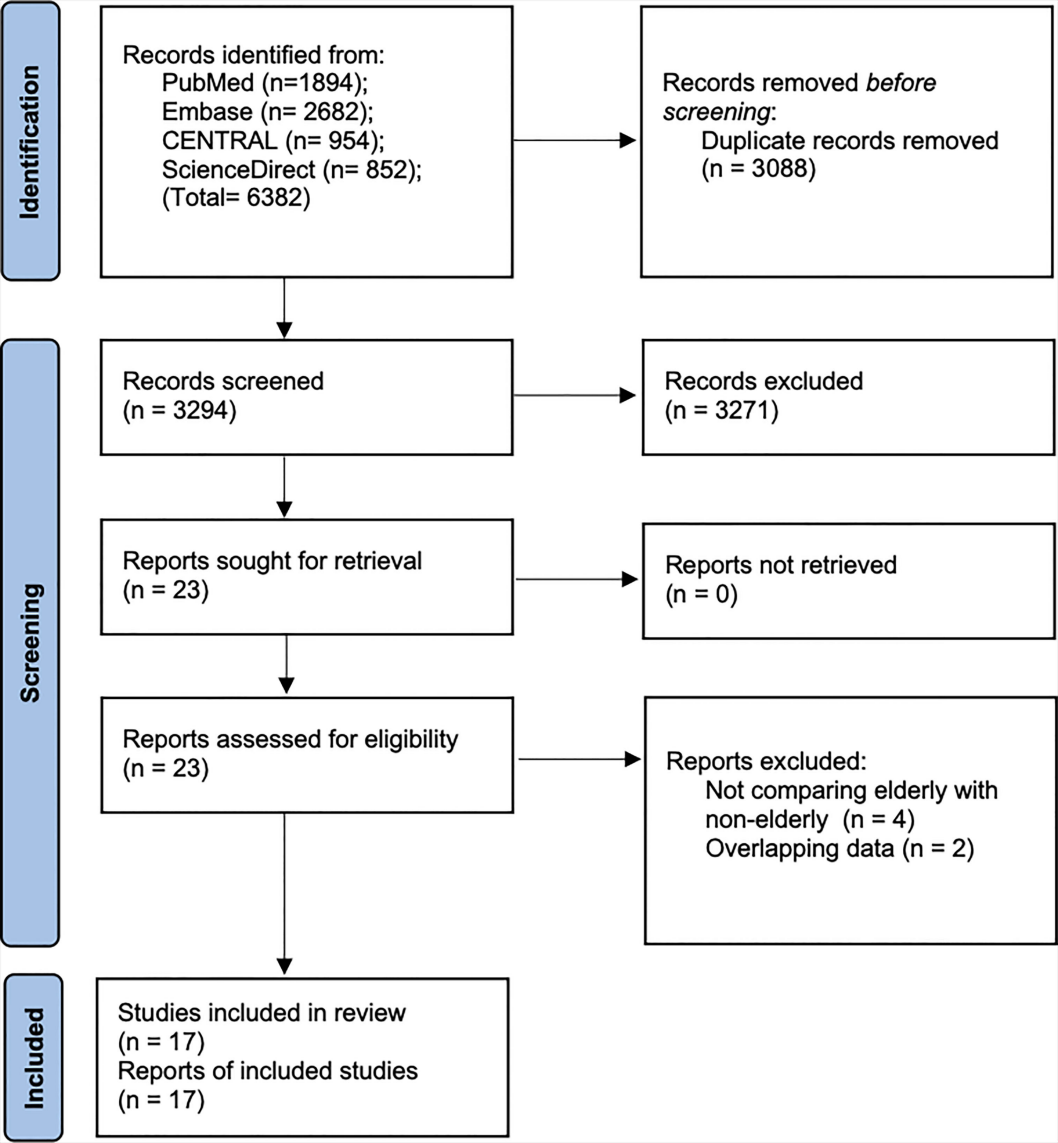
Study flow-chart.

A total of 6382 records were available after the literature search (**Figure 1**). After excluding duplicates, 3294 articles were examined by their titles and abstracts. 3271 studies were not found to be relevant to the review and hence excluded. 23 articles were screened by their full-texts and six (15–20) were excluded with reasons (**Table 1**). Finally, 17 cohort studies were found to be eligible for inclusion in this review (4, 10–12, 21–33).

**Table 1:** Details of excluded studies.

Study	Reason for exclusion
Abe 2012 (15)	Not comparing elderly vs non-elderly
Sumiyoshi 2017 (16)	Not comparing elderly vs non-elderly
Sekiguchi 2017 (17)	Not comparing elderly vs non-elderly
Chang 2020 (18)	Not comparing elderly vs non-elderly
Toya 2019 (19)	Overlapping data
Pyo 2017 (20)	Overlapping data

The data extracted from individual studies are presented in **Table 2**. All studies, except one (26), were retrospective in nature. The studies were published between 2005 to 2019. The majority of studies were conducted in Japan with just three of the included studies being conducted in China, Taiwan, and South Korea (one each). The definition early gastric cancer and indications of ESD were based mostly on the Japanese Gastric Cancer Association definitions [version 2 (35) or version 3 (36)] or either those defined by Gotoda et al. (34). The definition of the elderly population varied across studies with ≥75 years and <75 years being the most common classification used. Four studies classified the elderly as ≥80 years while one study classified them as ≥65 years. In the study of Yamaguchi et al. (27) and Watanabe et al. (4), three subgroups were subsumed to ≥80 years vs <80 years and ≥65 years vs <65 years respectively. The sample size of the elderly group ranged from 32 to 554 while in the non-elderly group it varied from 42 to 21860. None of the included studies carried out propensity score matching of the study groups. The NOS score of the included cohort studies varied from 6 to 8.

**Table 2:** Details of included studies.

Author/Year	Country	Definition of EGC	Groups (years)	Sample size	Mean Age (years)	Male gender (%)	CVS disease (%)	DM (%)	Use of AT/AC (%)	Ulcer finding (%)	Location U/M/L	Invasion depth IM/SM	Histological type D/UD	Tumor size (mm)	Lymphatic invasion (%)	NOS score
Yamaguchi 2019 (27)	Japan	JGCA version 3	>80	94 (107)^	83.9	69.1	24.5	NR	33	11.2	22/24/61	96/11	102/5	17.5	8.4	7
			65-79	266 (293)	72.3	71.4	18.8		22.2	9.9	52/82/159	253/40	281/12	15.6	6.5	
			<65	92 (101)	58.1	79.3	5.4		6.5	5	15/27/59	93/8	96/5	13.1	2	
Son 2019 (11)	South Korea	JGCA version NR	≥80	32	82.3	53.1	9.4	15.6	18.8	0	5/4/23	NR	32/0	NR	NR	6
			<80	406	64.5	64.8	8.4	16.5	18.2	1	23/64/319		404/2			
Okimoto 2019 (10)	Japan	JGCA version 3	≥80	108 (128)^	83.4	75.9	21.3	11.1	27.8	NR	16/35/77	114/14	125/3	16.5	3.9	8
			<80	425 (504)	69.6	72	13.6	10.8	16		87/143/274	456/48	489/15	16.5	3.4	
Watanabe 2017 (4)	Japan	JGCA version 3	≥85	43 (48)^	86	62.3	21	14	30.2	NR	13/15/20	40/8	46/2	NR	6.3	6
			65-84	511 (652)	75	68.9	13.3	15.7	24.1		122/272/258	564/88	634/18		6.7	
			≤64	161 (177)	60	83.2	10	13.4	11		37/73/67	164/13	170/7		2.3	
Otsuka 2017 (12)	Japan	JGCA version 3	≥80	64	84.2	68.7	35.9	31.2	6.3	NR	28/16/20	NR	63/1	17.5	NR	6
			<80	168	69.5	70.2	14.9	28	7.1		39/55/72		149/9	15.6		
Kato 2016 (26)	Japan	JGCA version 3	≥75	345 (421)^	80	69	11.9	19.4	25.2	13.3	77/210/134	386/35	408/13	17.5	NR	8
			<75	547 (641)	65	80.6	4.6	14.3	11.9	14.5	102/332/207	572/69	610/31	16.6		
Chinda 2015 (24)	Japan	NR	≥75	102 (109)^	79.2	63.7	21.6	10.8	30.4	NR	NR	NR	NR	23.5	NR	6
			<75	205 (209)	65.9	76.5	10.2	20.5	16.6					20.1		
Yang 2015 (25)	Taiwan	JGCA version 3	≥75	44	81.6	81.8	38.6	43.2	11.4	NR	0/28/16	37/7	44/0	22	NR	6
			<75	42	63.4	69	11.9	26.2	16.7		1/18/23	40/2	41/1	19.5		
Zhang 2014 (23)	China	Gotoda et al. (34)	≥75	46 (51)^	79	71.7	17.4	21.7	8.7	21.6	9/17/25	NR	NR	NR	NR	8
			<75	125 (136)	59.4	63.2	10.4	15.2	4.8	15.4	9/44/83					
Murata 2014 (22)	Japan	NR	≥80	5525	NR	65.5	5.5	12.2	7.7	NR	569/2801/2155	NR	NR	NR	NR	6
			<80	21860		76.2	2.8	11.8	4.1		1880/12001/7979					
Tokioka 2012 (21)	Japan	JGCA version 2	≥65	372	73.9	69.9	7.8	15.3	NR	NR	25/109/229	98.6	341/0	15.1	NR	7
			<65	143	57.7	82.5	0.7	6.3			23/45/74	96.5	139/4	14.5		
Toyokawa 2010 (33)	Japan	NR	≥75	200 (229)^	80	64	20	21	5.5	NR	54/76/98	158/28	NR	19	NR	8
			<75	314 (357)	66	75.5	11	15	1.6		93/141/122	245/41		18		
Isomoto 2010 (32)	Japan	JGCA version 2	≥75	260 (279)^	NR	72.4	NR	NR	NR	3.7	44/129/105	222/57	NR	18	NR	6
			<75	401 (434)	NR	79				13.3	73/209/149	369/65		18		
Shimura 2007 (28)	Japan	Gotoda et al (34)	≥75	41 (45)^	78	80.5	24.4	2.4	19.5	NR	6/25/14	NR	NR	16	NR	7
			65-74	41 (45)	70	70.7	12.2	2.4	12.2		7/20/18			15		
			<65	34 (35)	61	82.4	2.9	5.9	5.9		4/16/15			17		
Onozato 2007 (31)	Japan	JGCA version 2	≥75	93	79.8	53.8	NR	NR	NR	14.6	18/45/47	102/8	NR	22.8	NR	6
			<75	133	66	79.7				20.5	25/35/81	114/27		21.8		
Kakushima 2007 (29)	Japan	JGCA version 2	≥75	49	NR	NR	NR	NR	NR	NR	NR	NR	NR	NR	NR	6
			<75	135												
Hirasaki 2005 (30)	Japan	JGCA version 2	≥75	53	78.2	64.2	1.9	9.4	11	NR	NR	47/6	NR	12.2	NR	6
			<75	91	64.7	81.3	5.5	14	8.8			83/8		13		

^ figures in parenthesis indicates number of lesions.

CVS, cardiovascular disease; DM, diabetes mellitus; AT, antithrombotic; AC, anticoagulant; U, upper; M, middle; L, lower; IM, intramucosal; SM, submucosal; NOS, Newcastle-Ottawa scale; D, differentiated; UD, undifferentiated; NR, not reported; JGCA, Japanese Gastric Cancer Association.

### Primary Outcomes

#### En-Bloc Resection

**Figure 2:**
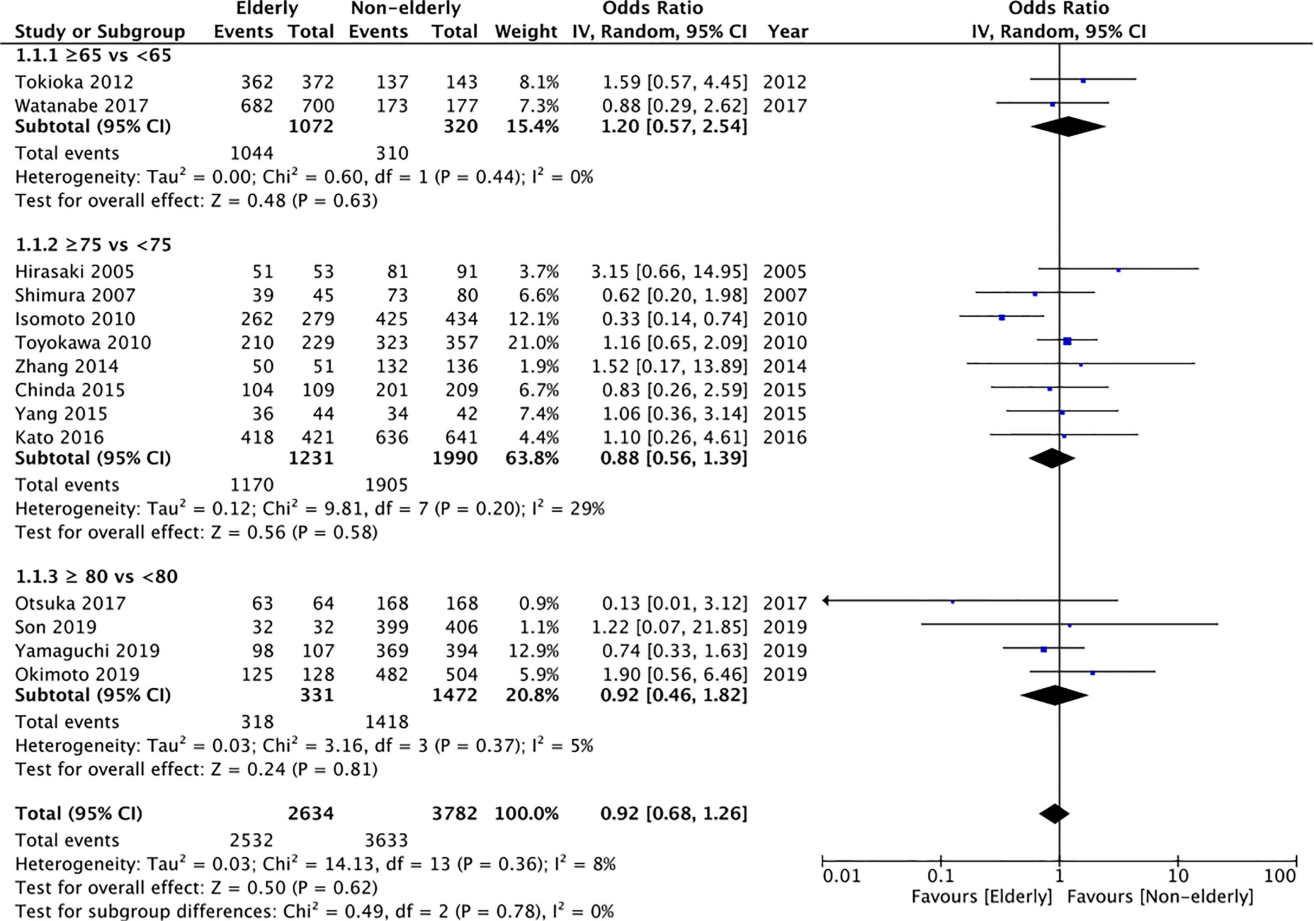
Meta-analysis of en-bloc resection rates between elderly and non-elderly patients with subgroup analysis based on definition of elderly subjects.

En-bloc resection rates were reported by 14 studies. Data of 2634 lesions in the elderly was compared with data of 3782 lesions in the non-elderly. Meta-analysis indicated no statistically significant difference in the en-bloc resection rates between the two groups (OR: 0.92 95% CI: 0.68, 1.26 I^2^ = 8% p=0.62) (**Figure 2**). The difference was non-significant even on subgroup analysis for ≥65 years vs <65 years (OR: 1.20 95% CI: 0.57, 2.54 I^2^ = 0% p=0.63), ≥75 years vs < 75 years (OR: 0.88 95% CI: 0.56, 1.39 I^2^ = 29% p=0.58), and ≥80 years vs <80 years (OR: 0.92 95% CI: 0.46, 1.82 I^2^ = 5% p=0.81). We found no evidence of publication bias on funnel plot (**Supplementary Figure 1**). However, the certainty of evidence was “very low” (**Supplementary Table 2**).

#### Histological Complete Resection

**Figure 3:**
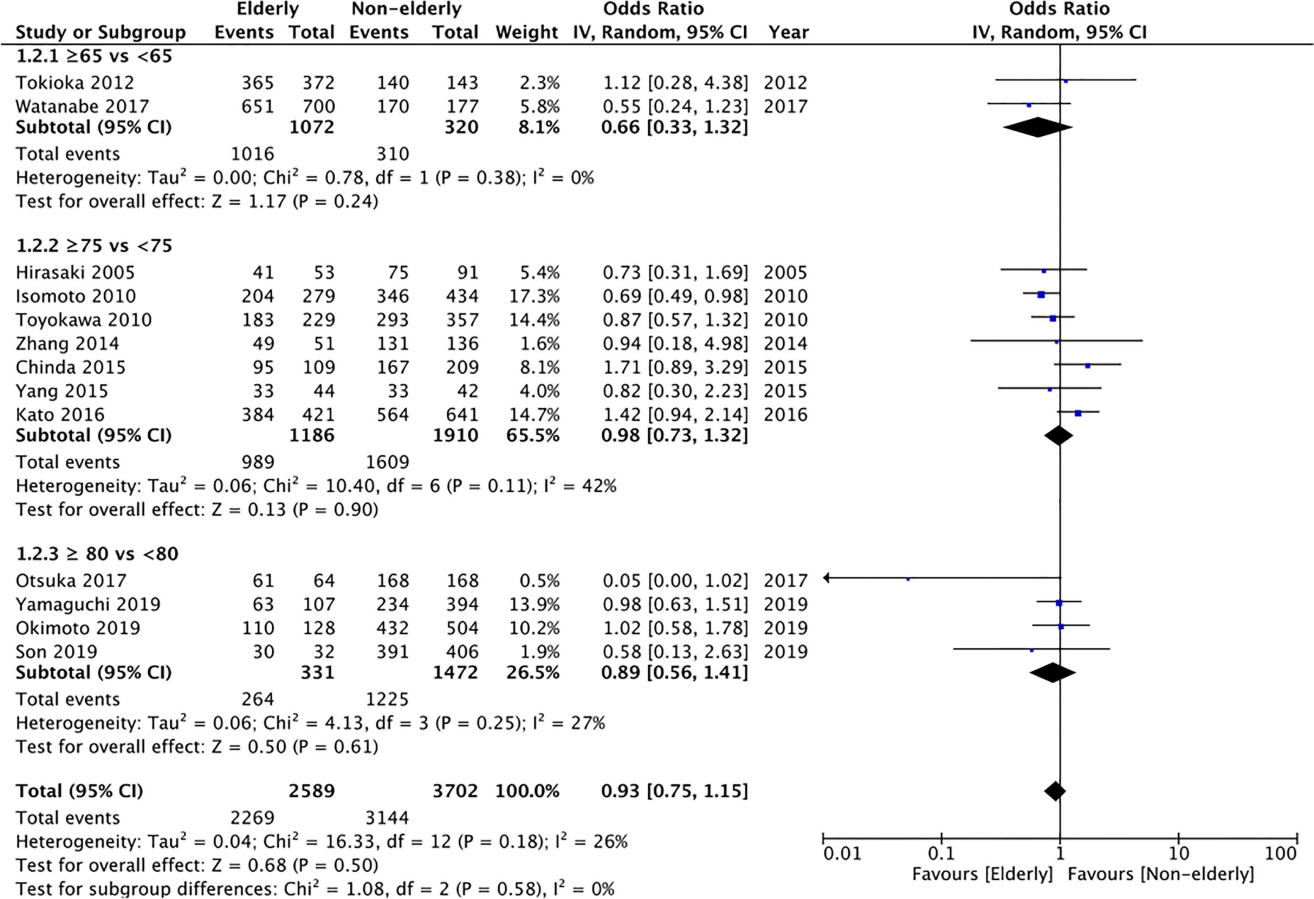
Meta-analysis of histological complete resection rates between elderly and non-elderly patients with subgroup analysis based on definition of elderly subjects.

13 of the 17 studies reported histological complete resection rates. Comparing 2589 lesions in the elderly group with 3702 lesions in the non-elderly group, we found no statistically significant difference in the histological complete resection rates between the two groups (OR: 0.93 95% CI: 0.75, 1.15 I^2^ = 26% p=0.50) (**Figure 3**). The difference remained non-significant on subgroup analysis for ≥65 years vs <65 years (OR: 0.66 95% CI: 0.33, 1.32 I^2^ = 0% p=0.24), ≥75 years vs < 75 years (OR: 0.98 95% CI: 0.73, 1.32 I^2^ = 42% p=0.90), and ≥80 years vs <80 years (OR: 0.89 95% CI: 0.75, 1.15 I^2^ = 26% p=0.50). There was no publication bias based on assessment of funnel plot (**Supplementary Figure 2**). On GRADE assessment the certainty of evidence was found to be “very low” (**Supplementary Table 2**).

### Secondary Outcomes

#### Perforation

**Figure 4:**
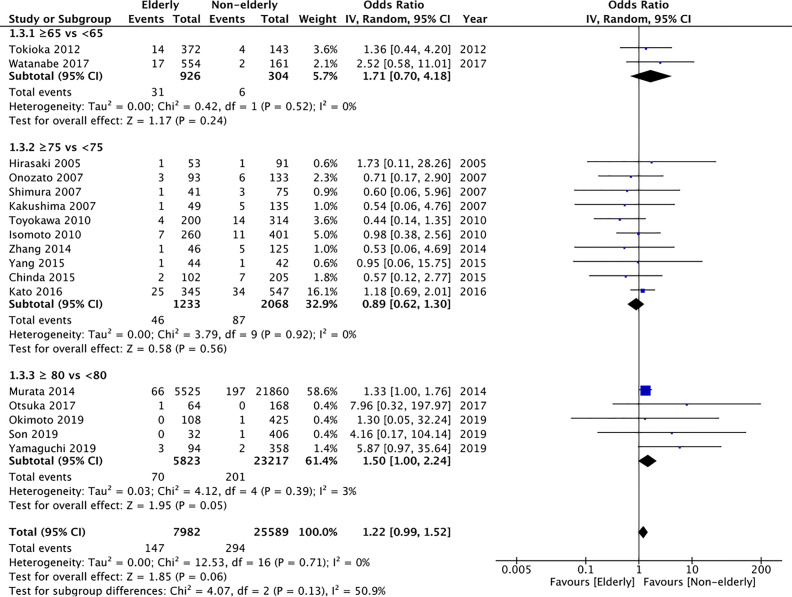
Meta-analysis of perforation rates between elderly and non-elderly patients with subgroup analysis based on definition of elderly subjects.

All included studies reported perforation rates. On pooled analysis, we found statistically non-significant but tendency of higher perforation rates in the elderly (OR: 1.22 95% CI: 0.99, 1.52 I^2^ = 0% p=0.06) (**Figure 4**). However, on subgroup analysis, there was a significantly increased risk of perforation in elderly patients aged ≥80 years as compared to patients <80 years (OR: 1.50 95% CI: 1.00, 2.24 I^2^ = 3% p=0.05); but no difference in the other subgroups of ≥65 years vs <65 years (OR: 1.71 95% CI: 0.70, 4.18 I^2^ = 0% p=0.24) and ≥75 years vs < 75 years (OR: 0.89 95% CI: 0.62, 1.30 I^2^ = 0% p=0.56). However, the certainty of evidence was “very low” (**Supplementary Table 2**).

#### Bleeding

**Figure 5:**
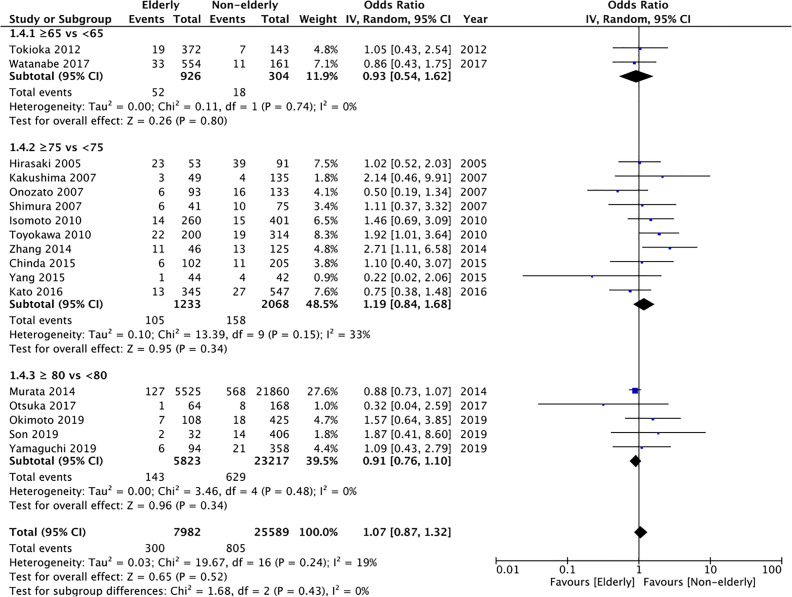
Meta-analysis of bleeding rates between elderly and non-elderly patients with subgroup analysis based on definition of elderly subjects.

Bleeding rates were reported by all studies. Meta-analysis demonstrated no statistically significant difference in the bleeding rates between the two groups (OR: 1.07 95% CI: 0.87, 1.32 I^2^ = 19% p=0.52) (**Figure 5**). The difference was non-significant even on subgroup analysis for ≥65 years vs <65 years (OR: 0.93 95% CI: 0.54, 1.62 I^2^ = 0% p=0.80), ≥75 years vs < 75 years (OR: 1.19 95% CI: 0.84, 1.68 I^2^ = 33% p=0.34), and ≥80 years vs <80 years (OR: 0.91 95% CI: 0.76, 1.10 I^2^ = 0% p=0.34). The certainty of evidence was found to be “very low” (**Supplementary Table 2**).

#### Pneumonia

**Figure 6:**
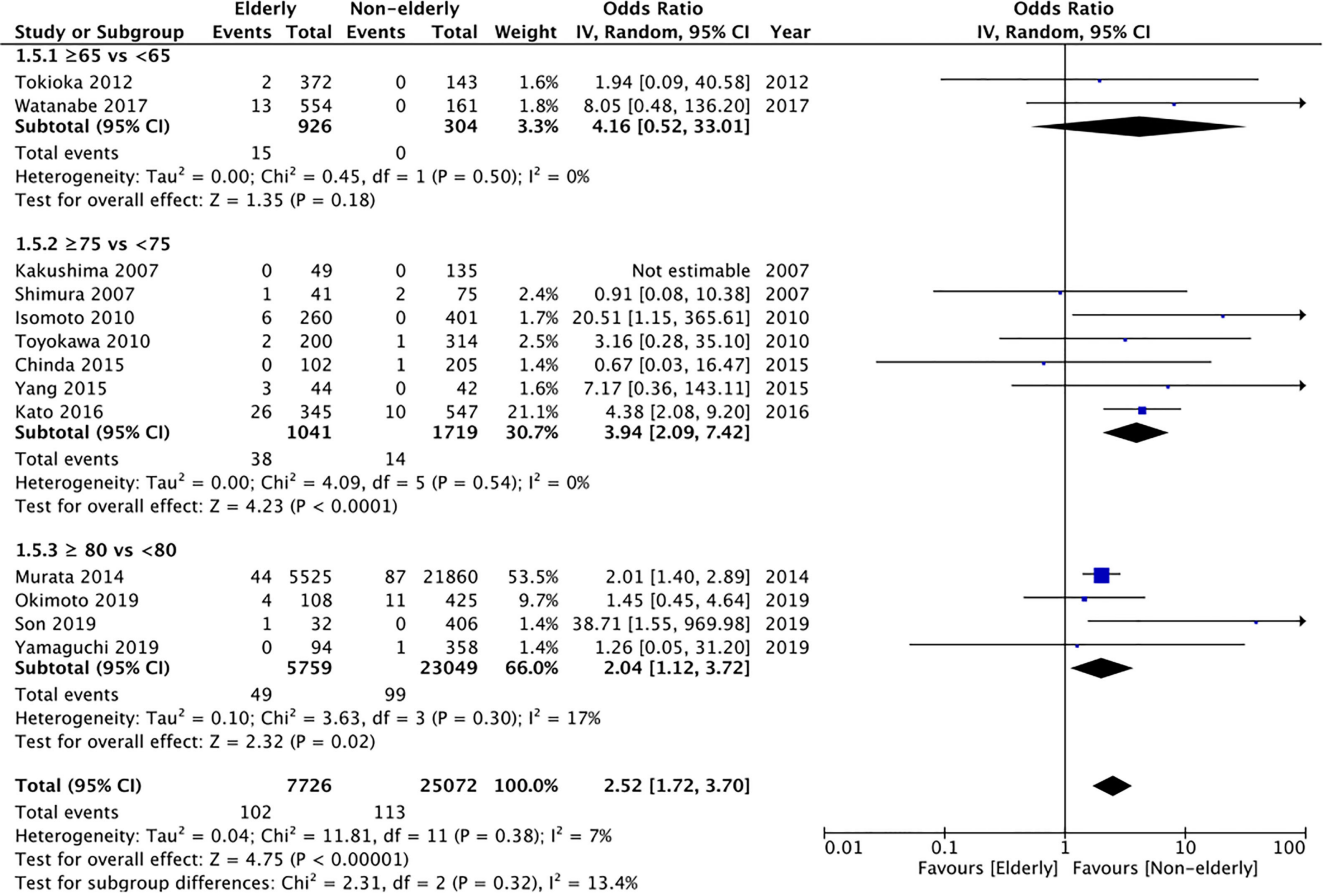
Meta-analysis of pneumonia rates between elderly and non-elderly patients with subgroup analysis based on definition of elderly subjects.

Data on pneumonia was reported by 13 studies. Pooled analysis indicated a statistically significantly increased risk of pneumonia in elderly patients (OR: 2.52 95% CI: 1.72, 3.70 I^2^ = 7% p<0.00001) (**Figure 6**). The incidence was significantly increased for the subgroup of ≥75 years vs < 75 years (OR: 3.94 95% CI: 2.09, 7.42 I^2^ = 0% p<0.0001) and ≥80 years vs <80 years (OR: 2.04 95% CI: 1.12, 3.72 I^2^ = 17% p=0.02) but non-significant albeit with a tendency of increased risk in the elderly for the subgroup of ≥65 years vs <65 years (OR: 4.16 95% CI: 0.52, 33.01 I^2^ = 0% p=0.18). On GRADE assessment the certainty of evidence was found to be “very low” (**Supplementary Table 2**).

#### Length of Hospital Stay

**Figure 7:**
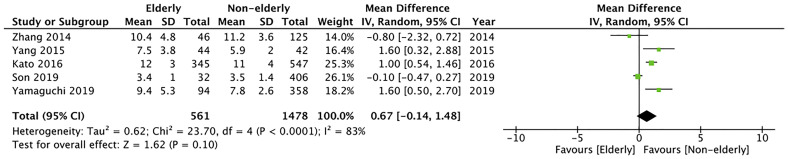
Meta-analysis of length of hospital stay between elderly and non-elderly patients.

Length of hospital stay was reported as mean and standard deviation only by five studies. Meta-analysis indicated no significant difference between the two study groups (MD: 0.67 95% CI: -0.14, 1.48 I^2^ = 83% p=0.10) (**Figure 7**). The certainty of evidence was found to be “very low” (**Supplementary Table 2**).

### Sensitivity Analysis

On the sequential exclusion of individual studies from the meta-analysis of resection rates and complications, we found no change in the direction of the result for any outcomes. All results were stable and maintained the significance of the overall effect. For all analyses with I^2^<50%, we also checked the pooled effect size using a fixed-effects model. However, there was no change in significance of any of the results (data not shown).

## Discussion

The elderly population is known to be at higher risk of cancer with most solid tumors being associated with older age. Data from the western population indicates that almost one-third of new cancer cases are detected in patients aged >75years and this figure may treble by 2040 (37, 38). Similarly, age is an important factor in the epidemiology of gastric cancer with tumors frequently detected in the elderly population (39). Guidelines for the management of gastric cancer are frequently derived from clinical trials conducted in a younger population with most studies excluding patients aged >75 years (40). In this context, it is important to differentiate the applicability and complications associated with gastric cancer treatment in this cohort of patients.

ESD has more or less replaced endoscopic submucosal resection (ESR) for the treatment of early malignancies of the gastrointestinal tract. ESR had technical limitations as lesions >15mm were prone to recurrence due to incomplete resection of the tumor (41). Studies have shown that ESD improves en-bloc and complete resection rates as compared to ESR but with a higher risk of complications due to technical challenges of the procedure (42). Since many physiological changes occur with increasing age including deterioration of organ function and changes in body composition, it is important to understand the efficacy of ESD for elderly patients (40).

For the primary outcomes of en-bloc resection rates and histological complete resection rates, our meta-analysis found no statistically significant difference between elderly and younger patients. Our results concur with the previous meta-analysis of Lin et al. (9) which also reported no statistically significant difference in en-bloc resection rates (OR: 0.98 95% CI: 0.56, 1.71) and histological complete resection rates (OR: 0.79 95% CI: 0.58, 1.07) between the two groups, albeit with an analysis of only six studies and a maximum sample size of 2146 patients. The current review was able to include up to 14 studies with a maximum sample of 6416 patients thereby significantly strengthening the validity of the results. Another important strength of our study was that we were able to differentiate between various subgroups of the elderly. The criteria for defining the elderly are known to differ in literature (43). Our analysis indicates that ESD can be carried out even in the “super-elderly” group of ≥80 years without any impact on en-bloc or histological complete resection rates. These rates are important as they are considered to be indicators to measure the oncological adequacy of ESD (36). However, our review was unable to decipher the long-term clinical outcomes of ESD in the elderly for want of data. In one of the included studies, Okimoto et al. (10) did not find any significant difference between overall survival and disease free-survival between patients aged ≥80 vs <80 years of age. On the other hand, Watanabe et al. (4) have reported higher mortality with early gastric cancer in the very elderly (≥85 years) and elderly (65-84 years) as compared to non-elderly patients. In addition to the contradictory results, at this point, it is also unclear if the overall survival is affected by differences in the clinicopathological characteristics of gastric cancer in the elderly group or if the higher comorbidity status plays a major role in influencing survival (40). Only further studies comparing elderly and non-elderly patients and assessing long-term survival can provide clarity on this subject.

Despite the minimally invasive nature of ESD, procedure-related adverse events are common especially perforation, bleeding, and pneumonia. The rates of perforation are known to vary from 1.2 to 9.6% with ESD for early gastric cancer (9, 44). In our review, we noted a perforation rate of 1.84% for the elderly group which was within the range described in the literature. Overall, the risk of perforation was not significantly higher but considering the 95% CI with the lower limit close to 1, there was a tendency of increased perforation rates in the elderly. Also, on subgroup analysis, we noted a 1.5 times increased risk of perforation in the super-elderly group of ≥80 years. Important to note is that many factors can influence perforation rates. A recent study by Ding et al. (44) has demonstrated that liver disease, upper location of the lesion, larger tumor size, submucosal invasion, longer operating time, gross lesion type, and piecemeal resection significantly affect perforation rates. Since the two cohorts in our study were not matched for baseline characteristics these factors may have influenced the outcome.

The number of comorbidities is known to increase with age many of which require anti-thrombotic and anticoagulant prophylaxis. While there has been no consensus on the effects of these drugs on bleeding rates with ESD, a recent meta-analysis suggests that regardless of continuation or discontinuation, antithrombotic drugs significantly increase the risk of delayed bleeding with ESD (45). Other lesion-related factors like lesion size, location on the lesser curvature, lesion morphology, histology, and ulcer finding also affect bleeding rates (46). In our review, we found no significant difference in the risk of bleeding with ESD between elderly and non-elderly patients. The non-significant results were noted even in the super elderly group of ≥80 years.

Elderly patients are prone to respiratory complications like pneumonia owing to the higher number of comorbidities and poor immune status. Indeed, a recent study has indicated that the Charlson comorbidity index of ≥3 is associated with an increased risk of respiratory-related complications in elderly patients undergoing ESD for early gastric cancer (47). Furthermore, lowered ability to expectorate post-procedure may also contribute to aspirational pneumonia. It is suggested that adequate suction may reduce the incidence of aspiration with ESD (9). In our analysis, we noted an increased risk of pneumonia in elderly patients irrespective of the cut-off age. The results were statistically non-significant for the subgroup of ≥65 vs <65 probably due to the limited number of studies in this analysis. But considering the 95% CI with an upper limit of 33, it is plausible that the risk of pneumonia is increased even with an age of ≥65 years.

The limitations of our review need to be specified. Foremost, it is important to note that all of the outcome variables can be influenced by several confounding factors. In the absence of baseline matching or multivariable-adjusted outcomes, the effect of several known and unknown confounding factors on the review outcomes cannot be negated. Since all included studies in the review were retrospective cohort in nature with inherent selection bias, the results should be interpreted with caution. On GRADE assessment of the outcomes, we found that the certainty of evidence was “very low” for all included outcomes. Secondly, the definition of elderly was not coherent in the included studies. While we attempted a subgroup analysis to better elucidate this difference, the variable definitions could have skewed the overall outcome. Thirdly, our review could not assess survival outcomes due to the non-availability of data. Long-term survival outcomes were reported only by Isomoto et al. (32), Okimoto et al. (10), and Watanabe et al. (4) wherein the elderly were defined as >75 years; >80 years and >65 years respectively. Considering the limited number of studies reporting the outcome with different definition of elderly, it was not feasible to conduct any pooled analysis for the outcome. Furthermore, such an analysis would have been biased, as it would include data of just three out of 17 studies. Fourthly, the majority of the studies included in our review were from a single country. The remaining studies too were from east Asia. This significantly limits the applicability of our results to western populations. Lastly, the definition of early gastric cancer and the indication for ESD did vary amongst the included studies. It needs to be highlighted that definition of early gastric cancer has broadened with time with Barreto et al. (48) now defining it as “An early gastric cancer is one that infiltrates the mucosa of the stomach without lymph node metastases. On biopsy or endoscopic specimen an early gastric cancer is <2 cm in maximum diameter, well differentiated, intestinal type, non-ulcerated, not depressed, located in the proximal stomach, and without infiltration beyond the mucosal layer or evidence of lympho-vascular invasion. On the surgical specimen an early gastric cancer is also without evidence on lymph node metastases from at least a D1 lymphadenectomy”. Future studies should use the expanded definition in order to present better evidence.

Despite these limitations, our review provides a comprehensive comparison of outcomes of ESD between elderly and non-elderly patients by pooling data from a large number of studies. Considering the small number of complications in individual studies, the meta-analysis provides pooled data with a significantly higher statistical power thereby strengthening the validity of the conclusions. The stability of the results on sensitivity analysis also lends support to the credibility of our results. Unlike the previous review (9), our study was also able to assess ESD outcomes in super-elderly patients. We believe the results of our study shall enable clinicians to make informed decisions and better anticipate outcomes in elderly and super-elderly patients with early gastric cancer.

To conclude, our study indicates that en-bloc and histological complete resection rates do not differ between elderly and non-elderly patients undergoing ESD for early gastric cancer. Elderly patients have a small tendency of increased risk of perforation with significantly increased rates in the super-elderly (≥80 years of age). The risk of pneumonia is significantly higher in elderly patients but the rates of bleeding do not differ. The certainty of evidence is “very low” and there is a need for high-quality studies taking into account confounding factors to enhance the quality of evidence.

## Data Availability Statement

The raw data supporting the conclusions of this article will be made available by the authors, without undue reservation.

## Author Contributions

JZ, ZS, and JL conceived and designed the study. JZ, ZS, and SG did literature search. JL and DH analyzed the data. JZ, ZS, and DH wrote the paper. JL and SG reviewed and edited the manuscript. All authors read and approved the final manuscript.

## Conflict of Interest

The authors declare that the research was conducted in the absence of any commercial or financial relationships that could be construed as a potential conflict of interest.

## Publisher’s Note

All claims expressed in this article are solely those of the authors and do not necessarily represent those of their affiliated organizations, or those of the publisher, the editors and the reviewers. Any product that may be evaluated in this article, or claim that may be made by its manufacturer, is not guaranteed or endorsed by the publisher.

## Supplementary Material

Supplementary Figure 1 Funnel plot of the meta-analysis of en-bloc resection rates between elderly and non-elderly patients.

Supplementary Figure 2 Funnel plot of the meta-analysis of histological complete resection rates between elderly and non-elderly patients
