# Supplementary figures and images for: Endoscopic Submucosal Dissection for Early Gastric Cancer in Elderly vs. Non-Elderly Patients: A Systematic Review and Meta-Analysis
Source: Front Oncol. 2022 Jan 13;11:718684. doi: 10.3389/fonc.2021.718684 (PMC8792970; doi:10.3389/fonc.2021.718684)

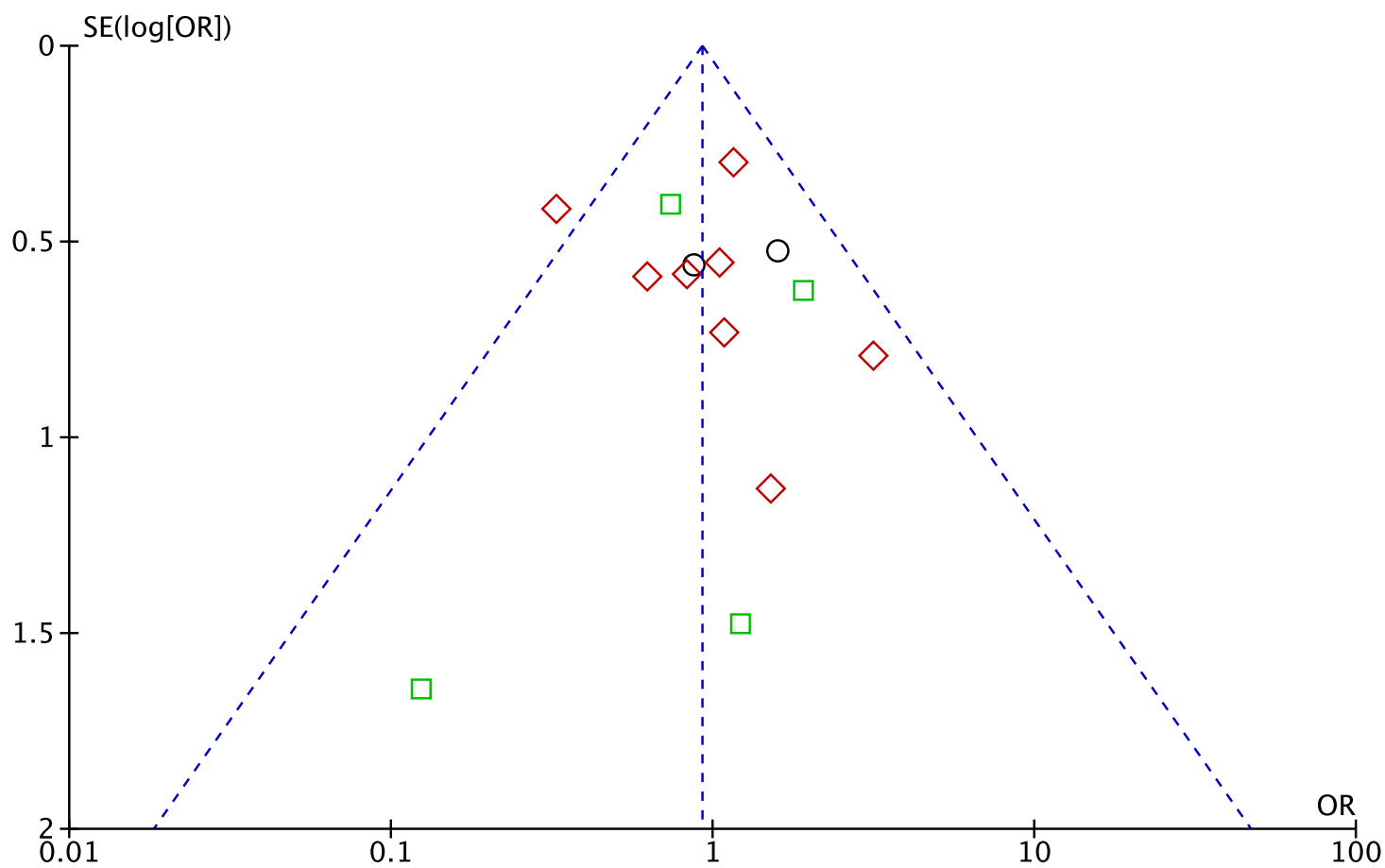

**Subgroups**

○ ≥65 vs <65    ◇ ≥75 vs <75    □ ≥80 vs <80

Supplement: Supplementary Figure 1 — Funnel plot of the meta-analysis of en-bloc resection rates between elderly and non-elderly patients. [file DataSheet_1.pdf]

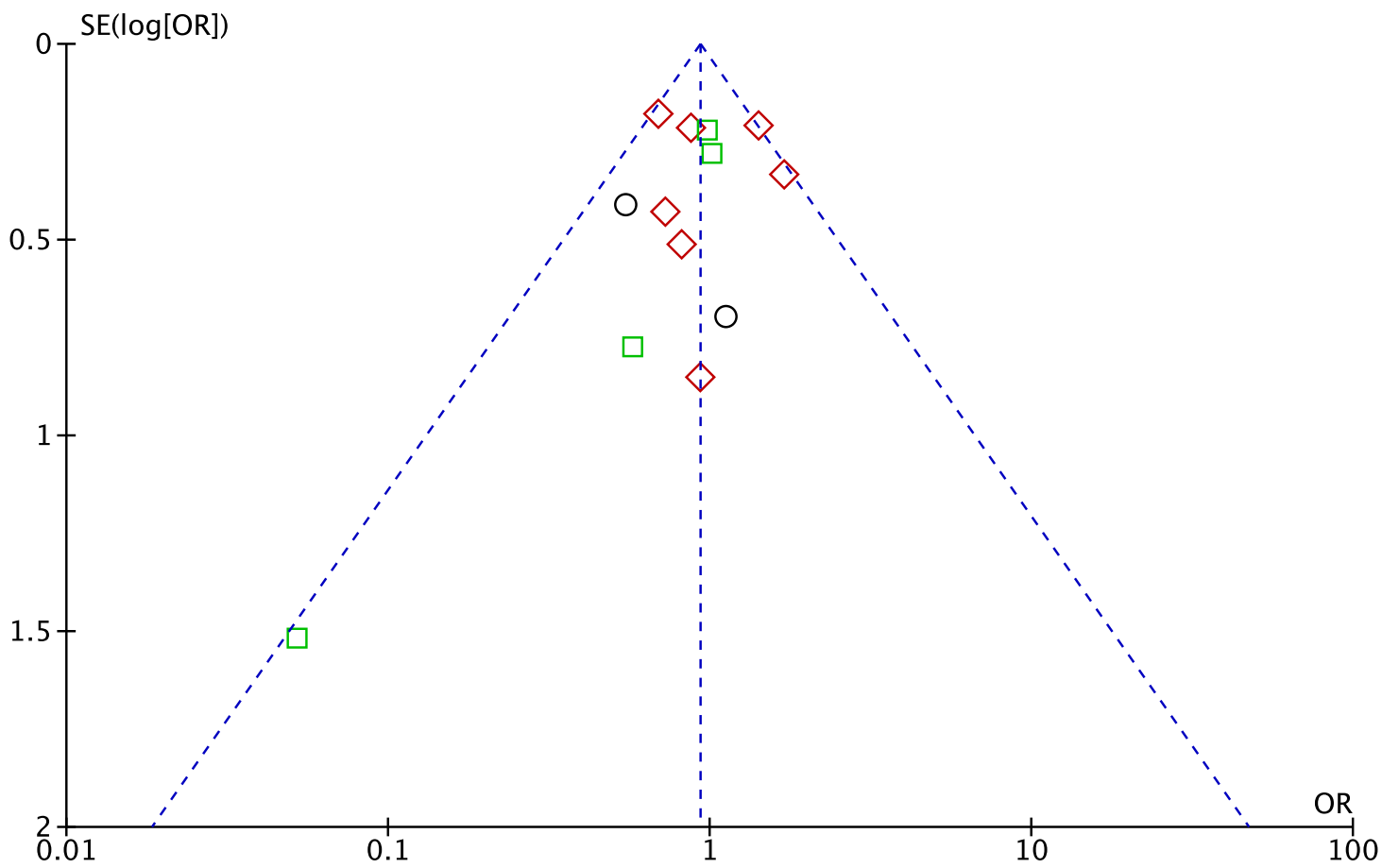

**Subgroups**

○ ≥65 vs <65    ◇ ≥75 vs <75    □ ≥80 vs <80

Supplement: Supplementary Figure 2 — Funnel plot of the meta-analysis of histological complete resection rates between elderly and non-elderly patients [file DataSheet_2.pdf]
