# Supplementary material for: Endoscopic Submucosal Dissection for Early Gastric Cancer in Elderly vs. Non-Elderly Patients: A Systematic Review and Meta-Analysis
Source: Front Oncol. 2022 Jan 13;11:718684. doi: 10.3389/fonc.2021.718684 (PMC8792970; doi:10.3389/fonc.2021.718684)
Supplement: Supplementary file 3 [file Table_1.docx]

Supplementary Table S1: Search strategy

| **Query** | **Search Details** |
| --- | --- |
| ((elderly) AND (endoscopic submucosal dissection)) AND (gastric cancer) | ("aged"[MeSH Terms] OR "aged"[All Fields] OR "elderly"[All Fields] OR "elderlies"[All Fields] OR "elderly s"[All Fields] OR "elderlys"[All Fields]) AND ("endoscopic mucosal resection"[MeSH Terms] OR ("endoscopic"[All Fields] AND "mucosal"[All Fields] AND "resection"[All Fields]) OR "endoscopic mucosal resection"[All Fields] OR ("endoscopic"[All Fields] AND "submucosal"[All Fields] AND "dissection"[All Fields]) OR "endoscopic submucosal dissection"[All Fields]) AND ("stomach neoplasms"[MeSH Terms] OR ("stomach"[All Fields] AND "neoplasms"[All Fields]) OR "stomach neoplasms"[All Fields] OR ("gastric"[All Fields] AND "cancer"[All Fields]) OR "gastric cancer"[All Fields]) |
| (((aged[Title/Abstract]) OR (older[Title/Abstract])) AND (endoscopic submucosal dissection)) AND (gastric cancer) | ("aged"[Title/Abstract] OR "older"[Title/Abstract]) AND ("endoscopic mucosal resection"[MeSH Terms] OR ("endoscopic"[All Fields] AND "mucosal"[All Fields] AND "resection"[All Fields]) OR "endoscopic mucosal resection"[All Fields] OR ("endoscopic"[All Fields] AND "submucosal"[All Fields] AND "dissection"[All Fields]) OR "endoscopic submucosal dissection"[All Fields]) AND ("stomach neoplasms"[MeSH Terms] OR ("stomach"[All Fields] AND "neoplasms"[All Fields]) OR "stomach neoplasms"[All Fields] OR ("gastric"[All Fields] AND "cancer"[All Fields]) OR "gastric cancer"[All Fields]) |
| ((geriatric) AND (endoscopic submucosal dissection)) AND (gastric cancer) | ("geriatric"[All Fields] OR "geriatrics"[MeSH Terms] OR "geriatrics"[All Fields]) AND ("endoscopic mucosal resection"[MeSH Terms] OR ("endoscopic"[All Fields] AND "mucosal"[All Fields] AND "resection"[All Fields]) OR "endoscopic mucosal resection"[All Fields] OR ("endoscopic"[All Fields] AND "submucosal"[All Fields] AND "dissection"[All Fields]) OR "endoscopic submucosal dissection"[All Fields]) AND ("stomach neoplasms"[MeSH Terms] OR ("stomach"[All Fields] AND "neoplasms"[All Fields]) OR "stomach neoplasms"[All Fields] OR ("gastric"[All Fields] AND "cancer"[All Fields]) OR "gastric cancer"[All Fields]) |
