# Supplementary material for: Endoscopic Submucosal Dissection for Early Gastric Cancer in Elderly vs. Non-Elderly Patients: A Systematic Review and Meta-Analysis
Source: Front Oncol. 2022 Jan 13;11:718684. doi: 10.3389/fonc.2021.718684 (PMC8792970; doi:10.3389/fonc.2021.718684)
Supplement: Supplementary file 4 [file Table_2.docx]

| **Supplementary Table 2: GRADE assessment of evidence** | | | | | | | | | | | |
| --- | --- | --- | --- | --- | --- | --- | --- | --- | --- | --- | --- |
| **Certainty assessment** | | | | | | | **Summary of findings** | | | | |
| **Participants (studies) Follow-up** | **Risk of bias** | **Inconsistency** | **Indirectness** | **Imprecision** | **Publication bias** | **Overall certainty of evidence** | **Study event rates (%)** | | **Relative effect (95% CI)** | **Anticipated absolute effects** | |
|  |  |  |  |  |  |  | **With Non-elderly** | **With Elderly** |  | **Risk with Non-elderly** | **Risk difference with Elderly** |
| **En-bloc-resection rate** | | | | | | | | | | | |
| 6416 (14 observational studies) | serious^a^ | not serious | not serious | not serious | none | ⨁◯◯◯ Very low | 3633/3782 (96.1%) | 2532/2634 (96.1%) | **OR 0.92** (0.68 to 1.26) | 961 per 1,000 | **3 fewer per 1,000** (from 17 fewer to 8 more) |
| **Histological complete resection rate** | | | | | | | | | | | |
| 6291 (13 observational studies) | serious^a^ | not serious | not serious | not serious | none | ⨁◯◯◯ Very low | 3144/3702 (84.9%) | 2269/2589 (87.6%) | **OR 0.93** (0.75 to 1.15) | 849 per 1,000 | **10 fewer per 1,000** (from 41 fewer to 17 more) |
| **Perforation** | | | | | | | | | | | |
| 33571 (17 observational studies) | serious^a^ | not serious | not serious | not serious | none | ⨁◯◯◯ Very low | 294/25589 (1.1%) | 147/7982 (1.8%) | **OR 1.22** (0.99 to 1.52) | 11 per 1,000 | **2 more per 1,000** (from 0 fewer to 6 more) |
| **Bleeding** | | | | | | | | | | | |
| 33571 (17 observational studies) | serious^a^ | not serious | not serious | not serious | none | ⨁◯◯◯ Very low | 805/25589 (3.1%) | 300/7982 (3.8%) | **OR 1.07** (0.87 to 1.32) | 31 per 1,000 | **2 more per 1,000** (from 4 fewer to 10 more) |
| **Pneumonia** | | | | | | | | | | | |
| 32798 (13 observational studies) | serious^a^ | not serious | not serious | not serious | none | ⨁◯◯◯ Very low | 113/25072 (0.5%) | 102/7726 (1.3%) | **OR 2.52** (1.72 to 3.70) | 5 per 1,000 | **7 more per 1,000** (from 3 more to 12 more) |
| **Length of hospital stay** | | | | | | | | | | | |
| 2039 (5 observational studies) | serious^a^ | not serious | not serious | not serious | none | ⨁◯◯◯ Very low | 1478 | 561 | - | The mean length of hospital stay was **0** | MD **0.67 higher** (0.14 lower to 1.48 higher) |

**CI:** confidence interval; **MD:** mean difference; **OR:** odds ratio

#### Explanations

a. downgraded due to lack of adjustment of confounding variables across majority studies
